# Effects of Soil Substrates and Microbial Inoculants on Earthworm-Mediated Modification of Soil Structure and Physicochemical Properties

**DOI:** 10.3390/biology15100735

**Published:** 2026-05-07

**Authors:** Hong Zhu, Xiaowei Cui, Wenyi Sheng, Yuzhi Wang, Xiao Wang, Pengcheng Zhu, Ning Du, Zhaojie Cui, Hongnian Chen, Jierui Dai, Lele Liu, Weihua Guo

**Affiliations:** 1 Key Laboratory of Ecological Prewarning, Protection and Restoration of Bohai Sea, Ministry of Natural Resource, School of Life Sciences, Shandong University, 72 Binhai Road, Qingdao 266237, China; 202332502@mail.sdu.edu.cn (H.Z.); shwy@sdu.edu.cn (W.S.); wangyuzhi@sdu.edu.cn (Y.W.); xiaowang_eco@163.com (X.W.); pczhu@sdu.edu.cn (P.Z.); ndu@sdu.edu.cn (N.D.); whguo@sdu.edu.cn (W.G.); 2 School of Municipal and Environmental Engineering, Shandong Jianzhu University, Jinan 250101, China; cuixw_1018@163.com; 3 School of Environmental Science and Engineering, Shandong University, Qingdao 266237, China; cuizj@sdu.edu.cn; 4 Shandong Provincial Lunan Geology and Exploration Institute (Shandong Provincial Bureau of Geology and Mineral Resources No. 2 Geological Brigade), Jining 272100, China; honianchen@126.com; 5 Shandong Institute of Geological Survey, No. 17 Jingshan Road, Lixia District, Jinan 250014, China

**Keywords:** bioturbation, aggregate stability, organic amendment, bioaugmentation, degraded land restoration

## Abstract

Earthworms play a crucial role in enhancing soil structure and nutrient cycling, especially in degraded lands. However, the interactive effects of soil amendments and microbial inoculants on their ecological functions remain underexplored. This study employed a full-factorial design to evaluate how different soil substrates (control soil, coal gangue-incorporated soil, organic fertilizer-incorporated soil, organic fertilizer surface-applied soil) and microbial inoculants (*Bacillus megaterium* and compound *Bacillus*) influence earthworm biomass and their engineering functions in soil. Our results demonstrated that the mean change in earthworm biomass in the control soil was −1.92 g, while the combined application of surface-applied organic fertilizer and compound Bacillus promoted earthworm growth, increasing the biomass change to 1.50 g, which was significantly higher than that in the control soil. Coal gangue addition did not adversely affect earthworms compared with the control soil. Furthermore, earthworm burrowing activity effectively improved soil structure and facilitated nutrient mixing. Notably, microbial inoculants affected soil electrical conductivity and large-aggregate content but had no significant effect on soil nutrients, while their interaction with soil amendments significantly affected only soil electrical conductivity. This study reveals that the co-application of organic fertilizer and earthworms can effectively improve soil nutrients and structure and that the regulatory effect of microbial inoculants on soil structure is substrate-dependent. These findings provide valuable insights for sustainable land management.

## Simple Summary

Healthy soil is needed to grow food and support nature, but many soils have become degraded. In this study, we tested whether adding different materials such as compost (organic fertilizer), a mining byproduct called coal gangue, and beneficial bacteria could help earthworms improve soil quality. We tested these materials alone and together in a controlled experiment. The results showed that earthworms grew best when compost was spread on the soil surface together with a mixture of beneficial bacteria. Under this treatment, earthworms created more large soil clumps and made the soil more stable. Adding coal gangue did not harm earthworms and also improved soil clumping, but it did not help earthworms grow in the short term. When compost was mixed fully into the soil, earthworms helped recycle nutrients like carbon and nitrogen, but they did not further improve soil structure. Bacteria alone had little effect on soil nutrients. In conclusion, the best way to restore degraded land is to use surface-applied compost together with earthworms and a mix of beneficial bacteria. Coal gangue can be added safely to improve soil structure. These findings offer practical, low-cost ideas for sustainable farming and land restoration.

## 1. Introduction

Degraded soils in agricultural and mining-affected regions face critical challenges including compaction [1,2], depletion of organic matter, declining fertility, and diminished microbial diversity [3,4]. These degradation processes directly undermine land productivity and weaken ecosystem functions [5], thereby threatening agricultural sustainability and environmental stability [6,7]. While soil organisms such as earthworms are known to play vital roles in maintaining soil structure and nutrient cycling, the interactive effects of organic amendments and microbial inoculants on these ecological functions in degraded soil contexts remain largely unexplored [8].

Long-term intensive cultivation is a primary cause of soil degradation [9]. As traditional soil amendments, organic fertilizers effectively enhance soil physicochemical properties while optimizing nitrogen and phosphorus concentrations [10]. These fertilizers facilitate comprehensive soil remediation through key mechanisms: reconstructing physical structures, modulating chemical properties, and stimulating biological activity [11]. In addition to traditional soil substrates such as organic fertilizers, coal gangue has great potential as solid waste in soil remediation [12,13]. Containing abundant silicon, aluminum, and iron, this material performs dual functions: neutralizing acidic soils and enhancing soil aeration and water retention via its porous structure. Coal gangue can be combined with aeolian sandy soil to formulate planting substrates that effectively promote the growth of *Kochia prostrata* while achieving dual benefits of waste reduction and ecological restoration [14]. When applied to soils, coal gangue simultaneously addresses waste recycling and soil quality improvement [15], representing a sustainable and economically viable remediation strategy [16].

However, despite these potential benefits, research on how coal gangue affects soil animals and microorganisms remains limited. Specifically, knowledge gaps exist regarding the effects of coal gangue on: (i) the survival, growth, and burrowing behavior of key soil engineers such as earthworms; and (ii) soil aggregation dynamics and aggregate stability, which are basic to soil structure. Furthermore, it remains unclear whether coal gangue exerts synergistic or antagonistic effects with soil microorganisms, particularly regarding their combined influence on soil biological processes and structure formation. Addressing these knowledge gaps is essential for evaluating the ecological implications of coal gangue application in soil restoration and for developing safe and effective strategies for remediating degraded lands.

Soil fauna constitutes a vital component of soil ecosystems, driving material cycling, energy flow, and key ecosystem functions [17]. Earthworms are recognized as “ecosystem engineers” due to their profound influence on soil physical, chemical, and biological properties through feeding, burrowing, and casting activities [18,19]. Their burrowing enhances soil porosity [20], while mucilaginous compounds in their casts act as binding agents, improving aggregate water stability [21]. The porous structure of casts facilitates gas exchange, and organic–mineral complexes within them enhance soil water-holding capacity [22].

Earthworm burrowing behavior is modulated by soil structural and physicochemical properties. For instance, co-application of organic fertilizer with coal gangue may increase porosity and facilitate burrowing, whereas large gangue particles could impede movement. Earthworm casts also possess unique nutrient-regulating properties, elevating nitrogen mineralization rates and available phosphorus content [23]. The study uses *Eisenia fetida*, an epigeic species typically associated with organic-rich environments rather than mineral soils. As a model organism, it reproduces quickly, is easy to cultivate, has strong tolerance, and is suitable for controlled microcosm experiments.

Furthermore, earthworm intestinal secretions and excreta supply abundant carbon sources and nutrients for microorganisms, particularly fostering beneficial microbial communities such as nitrogen-fixing and phosphorus-solubilizing bacteria [24]. The physical and chemical reactions between earthworms and the soil during earthworm activity directly affect the content of soil microorganisms and enzyme activity and indirectly affect soil nutrients [25]. In turn, microorganisms support earthworm growth and reproduction, while also influencing their digestive, immune, and other physiological functions [26,27]. The interactions between earthworms and other organisms, as well as soil microorganisms, serve as the biological foundation for maintaining the homeostasis of the soil ecosystem. These interactions play a crucial role in improving soil physical properties and regulating ecological processes such as soil carbon and nitrogen cycles.

Microorganisms play pivotal roles in maintaining soil health [28]. They sustain soil health by enhancing nutrient cycling, enriching essential nutrients, improving soil structure, and suppressing pathogens [29]. Combining microbial inoculants with organic amendments can create a more comprehensive soil remediation system [30]. Rhizobacterial inoculation, particularly when combined with organic amendments, significantly enhances the remediation of degraded semi-arid soils while promoting vegetation growth and restoration [31]. *Bacillus* strains are widely recognized for their ability to enhance nutrient availability through processes such as nitrogen fixation, phosphorus solubilization, and potassium mobilization. They also produce extracellular polymeric substances that contribute to soil aggregation and structural stability [32]. Additionally, as biocontrol agents, certain *Bacillus* strains are known to suppress soil-borne pathogens. Furthermore, their spore-forming capability ensures long-term survival and stability in soil environments, offering distinct advantages for both practical application and scientific research. The present study, however, focuses on their roles in nutrient cycling and soil structure improvement, rather than evaluating their function in pathogen suppression. When favored by suitable pH and high soil organic carbon, *Bacillus* strains proliferate in soil and promote macroaggregate formation through their hyphal networks and secretions [33]. A stable soil structure in turn creates favorable conditions for microbial colonization, reinforcing their beneficial effects [34]. Moreover, these bacteria can secrete substances such as organic acids to help maintain salt balance in saline soils, thereby reducing the impact of salinity on crop growth [35]. Studies have shown that the earthworm gut microbiome plays a crucial role in earthworm growth by enhancing nutrient digestion [36,37], stimulating metabolic efficiency, and promoting carbon assimilation through microbe-mediated decomposition and stabilization of organic matter [38]. Additionally, microorganisms in the earthworm gut and those in the soil environment jointly influence carbon metabolism processes in the soil [39].

To combat soil degradation and restore ecosystem functionality, we adopted a full-factorial design to investigate the integrated effects of soil substrates (organic fertilizer and coal gangue addition), earthworms (*Eisenia fetida*), and microbial inoculants (*Bacillus megaterium* and compound *Bacillus*) on soil physicochemical properties and earthworm growth. Although the individual benefits of organic amendments and earthworms have been well established, the interactive effects among these three factors, particularly the substrate-dependent role of microbial inoculants, remain poorly understood. Accordingly, we propose the following hypotheses: (i) soil substrates influence earthworm growth, thereby modulating their ecosystem-engineering roles; (ii) the effects of microbial inoculants on soil physicochemical properties and earthworm ecological engineering functions are influenced by soil substrates; and (iii) earthworm burrowing activity serves as a key manifestation of their engineering roles in soil structure formation and nutrient redistribution.

## 2. Materials and Methods

### 2.1. Experimental Design

The experiment was conducted from June to September 2023 in a controlled laboratory environment at Shandong University, China. The ambient temperature was maintained at 20–28 °C with a 12 h light/12 h dark photoperiod. The experimental soil was collected from Gudao Town, Hekou District, Dongying City, Shandong Province (detailed information on the soil sampling and physicochemical properties is provided in the Supplementary Methods and Table S1). The soil was classified as sandy loam, with a pH of 6.86 and an electrical conductivity of 363 µS·cm^−1^ (additional basic properties are listed in Table S1).

A full-factorial design with three factors was established: soil treatment (four levels), microbial inoculation (three levels), and earthworm addition (two levels), resulting in 24 treatment combinations. Polyethylene pots (height 15.4 cm, upper diameter 11.8 cm, lower diameter 8.0 cm) were filled with 0.8 kg of soil matrix (dry weight equivalent).

The control group (control) contained pure soil, while three treatments were established: T1 (coal gangue-incorporated soil), T2 (organic fertilizer-incorporated soil), and T3 (organic fertilizer surface-applied soil). All substrate soils were prepared by mixing soil with substrates at a 4:1 mass ratio (soil:substrate) (Table S1). None of the substrates were sterilized, but within each substrate, the initial physicochemical state was uniform. The organic fertilizer (collected from Jining City, China) contained 18.08% organic carbon, 1.01% total nitrogen, and 0.64% total phosphorus. Coal gangue (Cg) was obtained from Ling Shou County Huayao Mineral Products Processing Plant.

The microbial inoculants used in this study were *Bacillus megaterium* (Bm) and a compound *Bacillus* (CB) preparation containing *Bacillus amyloliquefaciens*, *Bacillus mucilaginosus*, and *Paenibacillus polymyxa*. Both inoculants had an initial concentration of 1.0 × 10^10^ CFU·g^−1^. For each experimental unit, 0.4 g of the original inoculant was suspended in 40 mL of sterile water and uniformly mixed into the soil, resulting in a final dose of 5.0 × 10^6^ CFU·g^−1^ soil. Control treatments received 40 mL of sterile water without inoculant.

Earthworms (*Eisenia fetida*) were sourced from the National High-Tech Industrial Development Zone (Yucheng) in Dezhou, Shandong Province. Individuals with an average fresh weight of 0.25 ± 0.05 g (sub-adult stage) were selected. For the earthworm treatments (Ew), 5 individuals were introduced at the beginning of the experiment, and another 5 were added after three weeks (10 per pot total), resulting in a final cumulative biomass of approximately 2.5 g·pot^−1^. The control treatments received no earthworms. The pots were covered with perforated lids to prevent escape while allowing gas exchange. Soil moisture was maintained at approximately 30% (*w*/*w*) by daily weighing and water replenishment. Other experimental details can be found in the Supplementary Methods.

### 2.2. Earthworm Biomass and Soil Trait Measurement

After the experiment, the surviving earthworms were identified and counted, carefully rinsed with distilled water, and weighed to determine their fresh weight. The biomass change (BC) was then calculated (final fresh weight − initial fresh weight). Subsequently, the earthworms were dried at 80 °C for 12 h until a constant weight was achieved, which was recorded as the dry weight (DW).

The soil was air-dried and analyzed for mean weight diameter (MWD), large-aggregate content (LAC), electrical conductivity (EC), total nitrogen (TN), total phosphorus (TP), and total organic carbon (TOC).

After the soil was air-dried, it was passed through a 1 mm (18-mesh) sieve, weighed (5.00 g), and transferred to a clean 50 mL centrifuge tube. Then, 25 mL of distilled water (free of carbon dioxide) was added. Three blank controls (containing only distilled water) were prepared. All samples and blanks were shaken at 25 °C and 180 rpm for 30 min, followed by 24 h of equilibration. The pH was measured using a portable pH meter (FE28-Meter, Mettler Toledo, Shanghai, China). The electrical conductivity (EC) was measured using a conductivity meter (FE38, Mettler Toledo, Shanghai, China).

Soil TOC was determined by oxidizing 0.1–0.3 g of air-dried soil (<0.25 mm, 80-mesh sieve) with 5 mL of 0.8 mol L^−1^ potassium dichromate (Sinopharm Chemical Reagent Co., Ltd., Shanghai, China) and 5 mL concentrated sulfuric acid (Sinopharm Chemical Reagent Co., Ltd., Shanghai, China). The mixture was heated at 170–180 °C for 5 min in an oil bath, and residual dichromate was titrated with 0.2 mol L^−1^ ferrous sulfate (Nanjing Chemical Reagent Co., Ltd., Nanjing, China) to calculate carbon content [40]. A correction factor of 1.1 was applied to account for incomplete oxidation, as recommended by the standard method. Soil TN was determined by digesting 0.1 g of soil with 8 mL concentrated sulfuric acid and a catalyst (3.0 g K_2_SO_4_, 0.2 g CuSO_4_) (Sinopharm Chemical Reagent Co., Ltd., Shanghai, China) in a graphite digester at 400 °C for 1 h [41]. The cooled digestate was diluted to 100 mL, and a 10 mL aliquot was analyzed by the Kjeldahl method (Model K1160, Hanon, Jinan, China). Soil TP was determined by transferring 3 mL of the digestate to a 50 mL volumetric flask, followed by the molybdenum blue colorimetric method using ascorbic acid (Sinopharm Chemical Reagent Co., Ltd., Shanghai, China) as the reductant, with absorbance measured at 700 nm [42]. Analytical accuracy and precision were ensured by method blanks, standard reference materials, and triplicate analyses, yielding TP recoveries of 95–105%.

After the soil was air-dried, the soil was screened by 5 mm sieve and 50 g of soil was weighed and soaked with water for 10 min. After this, the soil was soaked by a TPF-100 soil aggregate analyzer (Zhejiang Top Cloud-agri Technology Co., Ltd., Hangzhou, China) (soil screen size from top to bottom was 3 mm, 2 mm, 1 mm, 0.5 mm, 0.25 mm, 0.053 mm) and wet-screened for 2 min at a rate of 30 times/min. Soil of each particle size was transferred to an aluminum box, dried at 60 °C for 10 h to semi-dry and then at 105 °C for 12 h to constant weight, weighed, and the soil weight data of each particle size was collected to calculate the particle size and distribution of aggregates at all levels. Large-aggregate content (LAC) is the sum of soil mass percentages above 0.25 mm, and mean weight diameter (MWD) is the sum of the weight percentage of all measured aggregates multiplied by the mean diameter of this grain grade [43].

### 2.3. Statistical Analysis

The data were organized in Microsoft Excel 2019 and statistically analyzed using R v4.3.2. Three-way Analysis of Variance (ANOVA) was applied to assess the effects of all three factors (soil substrates, microbial inoculants, earthworms) on the soil structure and physicochemical properties. A two-way ANOVA was used to test the effects of soil substrates and microbial inoculants on earthworm growth. Post hoc comparisons were performed using Duncan’s multiple range test (*α* = 0.05). When the assumptions of normality or homogeneity of variances were violated, non-parametric methods were employed: the Kruskal–Wallis test followed by Dunn’s post hoc test with Benjamini–Hochberg correction, and different letters represented significant difference. Principal component analysis (PCA) was used to visualize the variation in multiple soil physicochemical properties (*n* = 24). A Pearson correlation matrix was calculated to quantify pairwise relationships among soil parameters, and the resulting *p*-values were adjusted for multiple comparisons using the Benjamini–Hochberg false discovery rate (FDR) correction. Effect sizes for each factor were calculated, and the results were visualized using forest plots generated in Open MEE 1.1.1 [44] to compare treatment effects across different soil parameters. All visualizations were created using R packages ggplot2 v3.4.2 and patchwork v1.1.2.

## 3. Results

### 3.1. Earthworm Biomass

**Figure 1:**
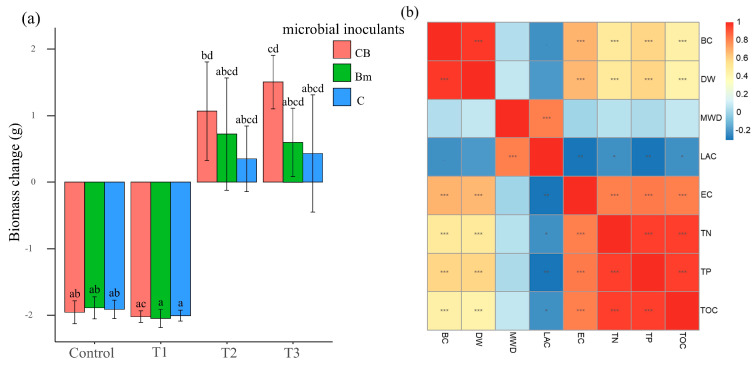
Variations in earthworm biomass in response to soil substrates and microbial inoculants. (**a**) Analysis of earthworm biomass change. Soil substrates: Control, coal gangue-incorporated soil (T1), organic fertilizer-incorporated soil. (T2), and organic fertilizer surface-applied soil (T3). All substrates were implemented under identical soil–water conditions to ensure comparability. Different colors represent microbial inoculant treatments. Blue is control (C), green is Bm, and red is CB. Different letters represented significant differences using a Kruskal–Wallis test followed by Dunn’s post hoc test with Benjamini–Hochberg correction; *α* = 0.05. (**b**) Pearson correlation analysis after FDR adjustment. Asterisks denote correlations that remained significant after correction (* *p*_adj_ < 0.05, ** *p*_adj_ < 0.01, *** *p*_adj_ < 0.001). BC, biomass change; DW, dry weight; LAC, large-agglomerate content; MWD, mean weight diameter; EC, electrical conductivity; TOC, total organic carbon; TN, total nitrogen; TP, total phosphorus.

The results showed that the mean change in earthworm biomass was −1.92 g in the control soil, −2.03 g in the coal gangue-incorporated treatment (T1), and 0.71 g in the organic fertilizer-incorporated treatment (T2). When organic fertilizer was surface-applied in combination with compound *Bacillus*, the change in earthworm biomass reached 1.50 g, which was significantly higher than that in the control group (*p* < 0.05) (Figure 1a). When earthworms were added, both the biomass and dry weight change in earthworms were significantly positively correlated with the soil EC and nutrients (Table S2), and negatively correlated with soil MWD and LAC, but not statistically significant (Figure 1b).

### 3.2. Soil Structure and Nutrient Characters

**Figure 2:**
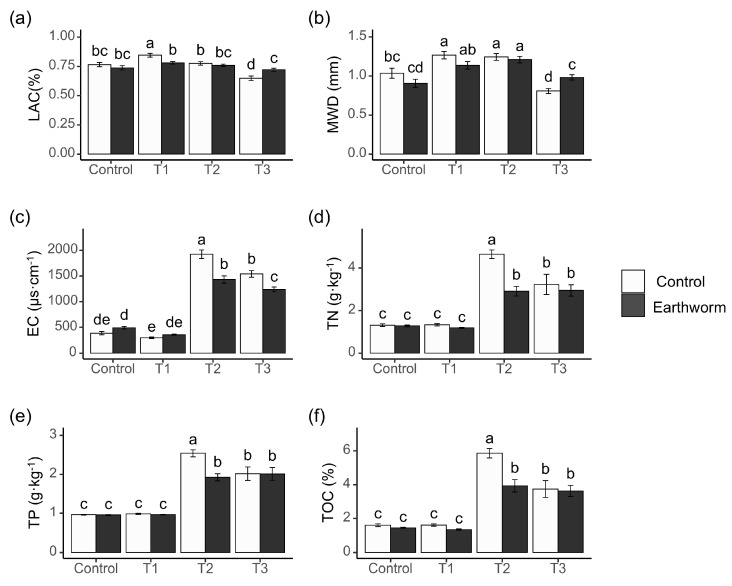
Differences in the effects of earthworm additions on the soil physicochemical properties under various soil substrate conditions. Soil substrates: control; coal gangue-incorporated soil (T1); organic fertilizer-incorporated soil (T2); organic fertilizer surface-applied soil (T3). White is no earthworms, black is added earthworms. Different letters represent significant differences; Duncan test, *α* = 0.05. (**a**) Analysis of soil large-aggregate content; (**b**) Analysis of soil mean weight diameter; (**c**) Analysis of soil electrical conductivity; (**d**) Analysis of soil total nitrogen; (**e**) Analysis of soil total phosphorus; (**f**) Analysis of total organic carbon.

The soil substrates and their interaction with earthworms significantly influenced the soil structure and physicochemical properties (Table S3). When organic fertilizer is applied to the soil surface, the vertical movement of earthworms significantly increases the soil’s MWD and LAC, which improves soil structure and enhances nutrient cycling. The application of organic fertilizer increased the soil TOC, TN, and TP content, but earthworms attenuated this enhancement. The addition of coal gangue improved the content of LAC and MWD, yet earthworms weakened its positive effect on soil LAC. Organic fertilizer mulching reduced the content of soil LAC and MWD, but the introduction of earthworms mitigated this effect (Figure 2).

Three-way ANOVA results showed that the soil structure and physicochemical properties were significantly affected by soil substrates and their interaction with earthworms (Table 1). Microbial inoculants significantly affected soil LAC and EC. Earthworms significantly affected soil nutrients but had no significant effect on soil LAC, and the interaction between soil substrates and microbial inoculants significantly affected EC. The interaction of the three factors did not have a significant effect on any measured variables, while the interaction between earthworms and microbial inoculants had no significant effect on the soil structure and physicochemical properties.

**Figure 3:**
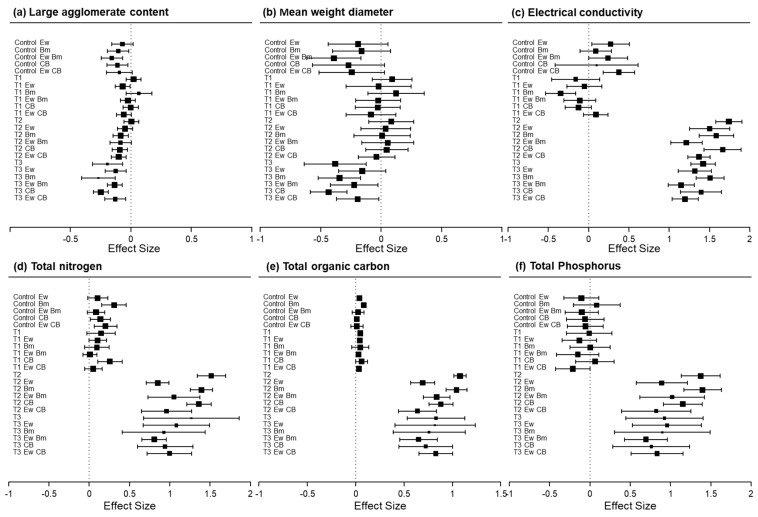
Mean effect sizes of treatments on soil structure (**a**,**b**) and physicochemical (**c**–**f**) properties. The effect sizes are calculated using the ln (response ratio). The size of the squares represents the weight of each treatment, and the error bars indicate the 95% confidence intervals. The X-axis represents the average effect sizes, and the Y-axis represents different treatment methods. The reference values for all metrics correspond to the soil structure and physicochemical properties under untreated conditions. Soil substrates: control; coal gangue-incorporated soil (T1); organic fertilizer-incorporated soil (T2); organic fertilizer surface-applied soil (T3); Ew, earthworm. Microbial inoculants: compound *Bacillus* (CB); *Bacillus megaterium* (Bm).

The experimental results revealed distinct treatment effects on soil properties under different substrate conditions. In the control soil, a significant reduction in soil LAC was exclusively observed in treatments involving compound *Bacillus* alone or compound *Bacillus* in earthworm-free systems, while other treatments showed no significant effects. Organic fertilizer application universally decreased soil LAC values across all treatment groups. Notably, when coal gangue was applied as substrate, none of the treatments significantly affected soil LAC (Figure 3a). Soil MWD was significantly affected by the treatments, with the effects varying among them. In the control soils, a significant reduction in MWD occurred only with *Bacillus megaterium*. The absence of a similar effect with compound *Bacillus* indicates functional specificity among microbial inoculants. The application of neither coal gangue substrates nor thoroughly mixed organic fertilizer significantly influenced soil MWD. Surface-applied organic fertilizer reduced soil MWD in all treatments, with the sole exception of the earthworm-only application (Figure 3b).

In the control soil, significant enhancement of soil EC was exclusively observed in treatments involving earthworms alone or earthworms combined with compound *Bacillus*, while other treatments showed no significant effects. This increase in electrical conductivity (EC) can be attributed to earthworm intestinal secretions directly introduced into the soil, which serve as electrolytes. The lack of a similar effect with *Bacillus megaterium* alone or compound *Bacillus* alone suggests that microbial inoculants, without earthworm activity, do not release sufficient ionic compounds to alter EC in nutrient-poor mineral soils. Organic fertilizer application universally increased EC values across all treatment groups. Notably, when coal gangue was applied as substrate, inoculation with *Bacillus megaterium* significantly reduced soil EC (Figure 3c).

In control soils, soil TN content increased only when earthworms were combined with either *Bacillus megaterium* or compound *Bacillus*. Specifically, the combination of earthworms and compound *Bacillus* resulted in a 22.5% increase in soil TN content. Coal gangue substrates enhanced soil TN exclusively in earthworm-inclusive treatments, while organic fertilizer universally promoted soil TN accumulation regardless of treatment or application method (Figure 3d). In control soils, treatment with *Bacillus megaterium* alone significantly increased soil TP. In contrast, when organic fertilizer was applied as substrate, all treatments showed a significant improvement in soil TP (Figure 3f). Soil TOC levels remained stable in both the control and coal gangue-incorporated soils; all organic fertilizer-amended treatments exhibited a significantly higher soil TOC (Figure 3e).

### 3.3. Soil Trait Principal Component Analysis

**Figure 4:**
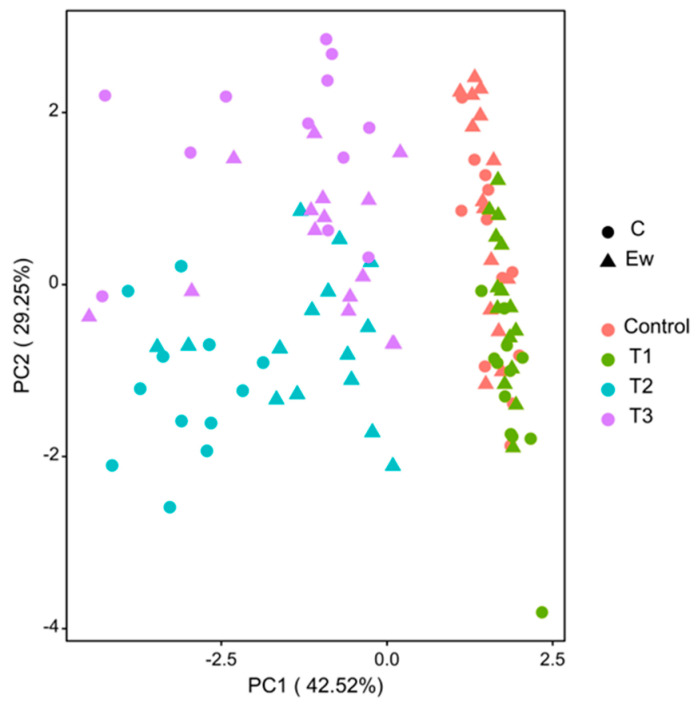
Principal component analysis of the effects of soil substrate and earthworm addition on the structure and nutrient properties of soil. In principal component analysis, C denotes control groups without earthworms, while Ew represents earthworm-inoculated treatments. The red points denote control soil, the green points denote coal gangue-incorporated soil, the blue points represent organic fertilizer-incorporated soil, and the violet points indicate organic fertilizer surface-applied soil.

The principal component analysis (PCA) of soil structure and physicochemical properties revealed that PC1 primarily represents soil EC, TN, TP, and TOC (Table S6), explaining 42.52% of the total variability (Figure 4). Two-way ANOVA shows that PC1 is significantly affected by the soil substrate, earthworm addition, and their interaction (Table S3). Higher PC1 values indicate lower levels of soil EC, TN, TP, and TOC, suggesting that the addition of organic fertilizers increased soil electrical conductivity and nutrient content. PC2 represents the soil structure indicators of soil LAC and MWD (Table S6), accounting for 29.52% of the total variability (Figure 4). PC2 is significantly affected by soil substrate and the interaction between the soil substrate and earthworms (Table S3). Higher PC2 values correspond to lower LAC and MWD values, indicating improved soil structure after the incorporation of organic fertilizers and coal gangue.

## 4. Discussion

The novelty of this study lies in its full-factorial design, which simultaneously manipulates three factors, including the substrate type, presence of earthworms, and the microbial inoculants. This design allowed us to disentangle not only the main effects of these factors but also their two-factor and three-factor interactions, combinations that have rarely been tested together. For each substrate type, a corresponding control was established to account for baseline differences in soil conditions. Unlike previous studies, we found that although organic fertilizer plays an important role in soil amelioration, its application alone did not significantly promote earthworm growth. However, when the microbial inoculant was co-applied with surface-applied organic fertilizer, earthworm growth was significantly enhanced. Furthermore, microbial inoculants significantly affected soil structure, whereas their influence on nutrient dynamics was substrate-dependent. These results reveal the context-dependent effects of microbial inoculants and the synergistic mechanisms among multiple factors, moving beyond the scope of traditional pairwise studies [45].

Earthworms redistribute soil nutrients vertically through bioturbation [46]. Specifically, they transport organic-rich surface soil to the subsurface, elevating organic carbon and available-nutrient levels there. Concurrently, their cast deposition enriches the topsoil with nutrients derived from deeper layers [47]. This bidirectional nutrient transport mechanism effectively mitigates vertical heterogeneity in soil nutrient distribution, resulting in a more balanced nutrient profile throughout the soil [48]. Studies have shown that under straw mulching conditions, earthworms can markedly enhance nutrient content, enzyme activity, and microbial biomass in the 0–10 cm soil layer [25]. The use of epigeic *Eisenia fetida* may bias our results toward organic fertilizer treatments, as this species is adapted to organic-rich environments. Compared with epigeic earthworms, the mechanisms by which different ecological groups of earthworms influence soil structure formation and nutrient redistribution through horizontal and vertical burrowing in mineral soils may differ fundamentally [49].

Therefore, in practical applications, it is necessary to validate the above conclusions and introduce endogeic earthworms for comparative studies to assess the impact of ecological type differences on soil amelioration effects. In experimental systems without organic fertilizer addition, we observed a decline in earthworm weight due to insufficient organic matter intake, a phenomenon also documented in other studies [50]. That study demonstrated a positive correlation between increasing organic fertilizer application rates and earthworm populations, with both total earthworm density and biomass showing proportional increases, particularly for *Eisenia fetida* in terms of both density and biomass [51]. Under organic fertilizer application, earthworms increase soil porosity and large-agglomerate content. When organic mulch is present, earthworms have sufficient food, and their feeding activities and epidermal mucus significantly improve soil aggregate structure [24]. The application of organic fertilizer improved earthworm survival rates and biomass, enabling them to better fulfill their role as “ecosystem engineers” [16].

The addition of organic fertilizers as a soil substrate promoted earthworm growth (Figure 1), thereby enhancing their role as ecosystem engineers and contributing to improved soil structure and nutrient status. The observed proliferation of immature earthworms in these treatments may indicate an active phase of organic matter transformation [26]. Through their feeding and excretion activities during growth, immature earthworms facilitate the initial decomposition and nutrient release of organic materials. This process lays the foundation for long-term soil fertility maintenance [52]. From an agricultural management perspective, protecting the entire life cycle of earthworms, particularly the immature stages, is essential to fully realize their long-term contributions to soil carbon cycling and soil health [53]. These findings highlight the potential importance of earthworm developmental stages in regulating soil organic matter dynamics, consistent with previous research. In contrast to the pronounced effects of organic substrate on earthworm growth, the addition of microbial inoculants did not significantly affect earthworm biomass. Although some variation was observed among different microbial treatments, these differences were not statistically significant.

Studies investigating various soil substrates have demonstrated that sustained organic fertilization significantly enhances soil macroaggregate content, increasing the proportion from 18% to 28% [54]. The proportion of macroaggregates and their organic carbon content are key determinants of soil organic carbon dynamics [34]. Increased macroaggregate content contributes to higher soil organic matter levels, and improving the mass fraction of macroaggregates is an effective strategy for enhancing soil fertility [55]. Moreover, higher soil organic matter promotes the binding and formation of soil aggregates, thereby stabilizing soil structure [56]. These findings are consistent with our observations that organic fertilization not only directly supports earthworm populations but also indirectly improves soil physical structure, creating a positive feedback loop for soil health.

However, it is important to note that the benefits of combined applications may be constrained by certain biophysical limits. Specifically, when organic matter inputs exceed a certain threshold, soil aggregates may become saturated, limiting further improvements in soil structure despite continued organic additions [57]. This saturation effect could explain why the combination of organic fertilizer and earthworm activity did not always yield additive benefits in our study. Additionally, a physical competition may exist between organic matter inputs and earthworm bioturbation for pore space and aggregate formation. While earthworms create macropores and ingest organic matter to form casts, excessive organic matter coating on soil particles or filling pore spaces could potentially interfere with earthworm burrowing activity and cast stability [58]. Understanding these threshold effects and competitive interactions is essential for optimizing the combined application of organic amendments and earthworms in soil remediation strategies.

Our study suggested that soil organic matter played a more dominant role than earthworms in promoting aggregate formation. This may be attributed to a structural saturation effect when exogenous organic matter is abundantly and uniformly incorporated into the soil. Under such conditions, it physically binds particles and forms aggregates, potentially overwhelming or masking the bioturbation effects of earthworms [56]. In this context, there may be physical competition between organic matter and bioturbation activity wherein organic matter physically occupies binding sites and pore spaces, limiting the relative contribution of earthworm activity to structural formation. Different application methods of organic fertilizer had varying effects on the soil physicochemical properties and structure (Figure 2). Compared to surface mulching, organic fertilizer-incorporated soil led to increases in earthworm biomass and soil carbon, nitrogen, and phosphorus content [46]. In our experimental design, the four soil substrates were established as distinct treatments prior to the introduction of earthworms and microbial inoculants. Consequently, the initial physicochemical differences among these substrates are inherent to the experimental setup and represent the target soil conditions to be compared, whereas within each substrate, the initial state was uniform. Notably, in the organic fertilizer-incorporated treatment, the fertilizer is homogeneously mixed throughout the topsoil, whereas in the surface-applied treatment, organic matter remains concentrated on the soil surface. This creates different microenvironments for earthworm activity, microbial decomposition, and nutrient release [59].

Coal gangue contains high levels of calcium carbonate, which can enhance soil physicochemical properties [60]. Our findings indicated that coal gangue addition did not exhibit toxic effects on earthworms, and organic fertilizer provided a favorable habitat for them. Therefore, in specific soil remediation scenarios, coal gangue can be used as a complementary measure alongside earthworms to achieve better soil improvement outcomes [61]. The increase in electrical conductivity observed in coal gangue-amended soils may be attributed to the release of soluble salts or base cations from gangue weathering. Electrical conductivity serves as a key indicator of soluble salt dynamics and microbial metabolic activity. Earthworm bioturbation and organic amendments can significantly alter EC by modifying soil solution composition and nutrient availability. In turn, EC influences earthworm behavior and microbial community structure, with optimal ranges supporting biological activity and extreme values causing stress. Thus, EC acts as both a response variable to treatments and a driving factor shaping soil biological functions [62].

Effect size analysis shows that in nutrient-poor mineral soils, microbial inoculants may temporarily disrupt aggregate stability by decomposing the limited organic matter. Specifically, the introduction of *Bacillus megaterium* can accelerate this decomposition, leading to a transient breakdown of macroaggregates, while the compound *Bacillus* did not show a similar effect. Notably, the presence of earthworms did not exacerbate this disruption. In contract, the physical restructuring of aggregates by earthworms partially offset the decomposition induced by microorganisms [63]. Coal gangue often contains elevated levels of soluble salts, *Bacillus megaterium* may promote salt leaching through enhanced microbial activity or facilitate microbial immobilization of ions, thus acting as a desalination agent [64]. This highlights that the functional role of microbial inoculants is substrate-dependent, as *Bacillus megaterium* exerts a beneficial salt-reducing effect specifically in saline-prone gangue environments. This synergistic effect likely arises from earthworm gut conditions that stimulate nitrogen-fixing bacteria, thereby enhancing biological N_2_ fixation [9].

Furthermore, *Bacillus megaterium* possesses phosphate solubilizing capacity, mobilizing fixed phosphorus in nutrient-poor mineral soils; however, under organic-rich conditions, the external phosphorus input from fertilizer overwhelms any additional solubilization effect, making treatment differences negligible. Finally, these results clearly demonstrate that soil organic carbon accumulation is dependent on exogenous organic matter input, as earthworms and microbial inoculants alone cannot increase the background carbon pool over the short term, given that they only regulate decomposition and turnover rates [65]. While the role of microbial communities in nutrient dynamics is well recognized [66], our study further demonstrates that under specific substrate conditions (unamended or coal gangue-amended soils), microbial inoculants can be associated with enhanced soil nutrient contents even in the absence of organic fertilizer (Figure 3). This finding underscores their potential to promote nutrient release and soil improvement across varying soil conditions [67].

Principal component analysis revealed that organic fertilizer addition significantly altered soil electrical conductivity and nutrient content. In the treatment with organic fertilizer-incorporated into the soil, the addition of earthworms negatively affected these indicators, whereas this negative effect was not evident in the surface-applied treatment. This is likely because earthworm bioturbation accelerates organic matter decomposition and nutrient mineralization [50]. In contrast, coal gangue addition had no significant effect on electrical conductivity or nutrient content, presumably due to its slow weathering and limited ion release during the three-month experimental period, as well as its inherently low soluble-nutrient content. The degree of physical mixing between the organic fertilizer and soil, determined by the application method, critically shaped the soil structure. Incorporation facilitated intimate organo-mineral binding, leading to markedly improved soil structure. The limited physical mixing under surface application resulted in weaker aggregate formation [68]. When organic fertilizer is surface-applied, the addition of earthworms helps to mix the organic fertilizer with the soil, promote the formation of biopores, and enhance aggregate stability, thereby effectively improving soil structure [19].

Therefore, in practical soil remediation efforts, native earthworm populations can be introduced based on specific soil environmental conditions. Our findings underscore the critical role of organic fertilizer in enhancing the ecological functions of earthworms [69]. Furthermore, the application of functional microbial inoculants can improve the soil’s structure and physicochemical properties, while their synergistic interaction with earthworms leads to more effective restoration [70]. Based on these insights, future applications should consider integrating a targeted proportion of coal gangue to improve soil structure, along with organic fertilizer to increase organic matter. This combined approach establishes a favorable habitat for earthworms and microorganisms, and the synergy of these measures ultimately contributes to successful soil remediation [71].

Our study demonstrates that the combined application of earthworms, organic amendments, and microbial inoculants can improve soil physicochemical properties. Here, we primarily used earthworm biomass as an indicator, but earthworm reproductive capacity, cast production, and soil microbial diversity are also key indicators for assessing earthworm ecological functions [72]. However, the present study does not include any direct measurements such as microbial biomass, enzyme activity, or community composition. Consequently, no microbial functional or community analyses were conducted, and the mechanistic conclusions remain hypothetical. Future research should integrate microbial sequencing to further elucidate how multiple factors collectively influence the soil amelioration process [73]. In addition, functional analyses are needed to establish mechanistic links between the treatments and the observed changes in nutrient availability. Moreover, plants play an important role in soil amelioration. Future research should introduce plant systems to study plant–soil interactions, focusing on how plants respond to changes in the soil environment induced by treatments such as earthworms, microbial inoculants, and organic materials, and how this response in turn feedbacks to affect soil aggregate formation, nutrient cycling, and carbon sequestration, thereby providing aboveground–belowground integrated evidence for a comprehensive explanation of soil amelioration mechanisms [74].

The experiment was conducted over approximately three months, which is relatively short for assessing soil structural dynamics, aggregation processes, and microbial interactions. Therefore, it captures only the immediate effects of the treatments and cannot reflect long-term dynamics, including earthworm population fluctuations or the persistence of microbial inoculants [75]. To address these gaps, long-term field trials under realistic conditions are required to further validate the effectiveness and ecological safety of these combined amendment strategies [64]. Such efforts will not only deepen our mechanistic understanding of earthworm–microbe–substrate interactions in soil remediation but also enhance their practical applicability in sustainable land management.

## 5. Conclusions

Organic fertilizer has been confirmed as a highly effective substrate for soil amelioration. The combined application of surface-applied organic fertilizer and the compound *Bacillus* promoted earthworm growth, thereby enhancing the engineering functions of earthworms. In organic-amended soils, earthworm burrowing behavior not only optimizes soil structure but also facilitates nutrient cycling. Although coal gangue contributes to structural improvement, it does not significantly promote earthworm growth over the short experimental period. In contrast, microbial inoculants can modulate soil electrical conductivity and macroaggregate content, yet they do not induce significant changes in soil nutrient status, indicating that the effects of microbial inoculants are limited but context-dependent.

This controlled experiment confirms that organic fertilizer, as a key substrate for soil restoration, acts synergistically with earthworms to optimize soil structure and nutrient cycling, and that the expression of earthworm ecological functions is distinctly substrate-dependent. Furthermore, the results also validate the feasibility of integrating coal gangue into multi-faceted rehabilitation strategies.

## Figures and Tables

**Table 1:** Three-way ANOVA on the main and interactive effects of the soil substrates, microbial inoculants, and earthworms on the soil physicochemical properties.

Trait	F (S)	F (M)	F (Ew)	F (S * M)	F (S * Ew)	F (M * Ew)	F (S * M * Ew)
MWD	24.35 **	2.65	0.77	0.70	4.67 **	0.50	0.63
LAC	24.50 ***	3.77 *	0.74	1.9	8.25 **	1.31	0.21
EC	392.76 ***	3.75 *	21.99 ***	2.46 *	18.37 ***	1.14	0.51
TN	64.05 ***	0.63	12.26 ***	0.99	6.55 ***	0.57	0.56
TP	89.84 ***	0.60	5.24 *	1.07	4.65 **	0.39	0.41
TOC	72.35 ***	1.00	9.95 **	0.82	5.06 **	0.39	0.27

Asterisks next to values represent significant differences (* *p* < 0.05, ** *p* < 0.01, *** *p* < 0.001). S, soil substrate; M, microbial inoculants; Ew, earthworm. MWD, mean weight diameter; LAC, large-aggregate content; EC, electrical conductivity; TN, total nitrogen; TP, total phosphorus; TOC, total organic carbon. Details of the three-way ANOVA are provided in the Supplementary Methods (Tables S4 and S5).

## Data Availability

The data will be made available on request.

## Supplementary Materials

The following supporting information can be downloaded at https://www.mdpi.com/article/10.3390/biology15100735/s1, Supplementary Methods, Table S1: Soil structure and physicochemical properties of four types of soil substrates; Table S2: Two-way ANOVA results of the effects of soil substrates (S) and microbial inoculants (M) on the earthworms and soil’s structure and physicochemical properties; Table S3: Two-way ANOVA results of the effects of soil substrates (S) and earthworms (Ews) on soil structure and physicochemical properties; Table S4: Effects of soil substrates, microbial inoculants, and earthworms on soil physicochemical properties (mean ± SD). Table S5: Three-way ANOVA (partial η^2^) results for the effects of soil substrates, microbial inoculants, and earthworms on soil structure and physicochemical properties; Table S6: Principal component analysis of the effects of soil substrates and earthworm addition on the structure and nutrient properties of soil.

## Abbreviations

The following abbreviations are used in this manuscript:

T1	coal gangue-incorporated soil
T2	organic fertilizer-incorporated soil
T3	organic fertilizer surface-applied soil
Ew	earthworm
CB	compound *Bacillus*
Bm	*Bacillus megaterium*
S	soil substrate
M	microbial inoculants
BC	biomass change
DW	dry weight
MWD	mean weight diameter
LAC	large-aggregate content
EC	electrical conductivity
TN	total nitrogen
TP	total phosphorus
TOC	total organic carbon
