# Supplementary material for: Effects of Soil Substrates and Microbial Inoculants on Earthworm-Mediated Modification of Soil Structure and Physicochemical Properties
Source: Biology (Basel). 2026 May 7;15(10):735. doi: 10.3390/biology15100735 (PMC13203635; doi:10.3390/biology15100735)
Supplement: Supplementary file 1 [file biology-15-00735-s001.zip › biology-4268244-supplementary.pdf]

Supplementary Methods

The experimental soil was collected from Gudao Town, Hekou District, Dongying City, Shandong Province (37°82′ N, 118°75′ E), located in the Yellow River Delta. This region belongs to a warm temperate semi-humid continental monsoon climate zone, with light loam being the predominant soil texture. The soil was taken from the 0-30 cm surface layer of farmland, air-dried, passed through a 2 cm sieve, and mixed after removing large debris and plant roots. The soil has an organic carbon content of 1.49%, total nitrogen of 0.11%, and total phosphorus of 0.089%, indicating relatively poor soil nutrient levels. Coal gangue (Cg) was obtained from Ling Shou County Huayao Mineral Products Processing Plant. Its main components include SiO_2, Al_2O_3, Fe_2O_3, CaO, MgO, TiO_2, P_2O_5, K_2O, and Na_2O. The heavy metal content of the coal gangue was lower than the limits set by the national soil pollution risk control standard for agricultural land (GB15618-2018). The organic fertilizer (collected from Jining City, China) contained 18.08% organic carbon, 1.01% total nitrogen, and 0.64% total phosphorus

The microbial inoculants used in this study were *Bacillus megaterium* (Bm) and a compound *Bacillus* (CB) preparation containing *Bacillus amyloliquefaciens*, *Bacillus mucilaginosus*, and *Paenibacillus polymyxa*. Both inoculants had an initial concentration of 1.0×10^{10} CFU·g^{-1}. For each experimental unit, 0.4 g of the original inoculant was sus-pended in 40 mL of sterile water and uniformly mixed into the soil, resulting in a final dose of 5.0×10^{6} CFU·g^{-1} soil. Control treatments received 40 mL of sterile water with-out inoculant.

Earthworms (*Eisenia foetida*) were sourced from National High-Tech Industrial Development Zone (Yucheng) in Dezhou, Shandong Province. After a 14-day acclimation period in the laboratory, the earthworms were cleaned, placed in sterile culture dishes for 24 hours to clear bacterial spores from their bodies, weighed, and then added to the experimental system

Table S1 Soil Structure and Physicochemical Properties of Four Types of Soil Substrates.

Substrate Type	(Soil: Substrate)	LAC (%)	MWD (mm)	EC (μS/cm)	TN (g/kg)	TP (g/kg)	TOC (%)
Control	soil	0.819±0.043	1.187±0.194	363.225±55.004	1.118±0.092	0.930±0.022	1.574±0.323
coal gangue -incorporated soil	4:1 (soil: coal gangue)	0.837±0.034	1.299±0.060	309.600±81.441	1.292±0.204	0.975±0.032	1.560±0.322
organic fertilizer -incorporated soil	4:1 (soil: Organic fertilizer)	0.822±0.024	1.291±0.129	2070.500±164.838	5.084±0.836	2.735±0.179	6.216±0.802
organic fertilizer surface -applied soil.	4:1 ((soil: Organic fertilizer)	0.675±0.084	0.811±0.172	1504.000±119.331	3.972±2.397	3.134±0.652	3.975±1.797

Note: MWD, mean weight diameter; LAC, large aggregate content; EC, electrical conductivity; TN, total nitrogen; TP, total phosphorus; TOC, total organic carbon

Table S2 Two-way ANOVA results of the effects of soil substrates (S) and microbial inoculants (M) on earthworms and soil structure and physicochemical properties.

Trait	*F*(S)	*F*(M)	*F*(S*M)
BC	32.31***	0.84	0.38
DW	27.72***	0.27	0.88
MWD	9.24***	0.74	0.52
LAC	3.76*	0.59	0.97
EC	180.14***	5.76**	2.06
TN	29.73***	0.14	0.85
TP	36.57***	0.03	0.94
TOC	29.26***	0.19	0.60

Note: Asterisks values represent significant differences (* $P < 0.05$, ** $P < 0.01$, *** $P < 0.001$). S, soil substrates; M, microbial inoculants. BC, biomass change; DW, dry weight; MWD, mean weight diameter; LAC, large aggregate content; EC, electrical conductivity; TN, total nitrogen; TP, total phosphorus; TOC, total organic carbon.

Table S3 Two-way ANOVA Results of the Effects of Soil Substrates (S) and Earthworms (Ew) on Soil Structure and Physicochemical Properties

Trait	*F* (S)	*P* (S)	*F* (Ew)	*P* (Ew)	*F* (S*Ew)	*P* (S * Ew)
MWD	24.779	**< 0.001**	0.784	**0.378**	4.746	**0.004**
LAC	22.938	**< 0.001**	0.696	**0.406**	7.724	**< 0.001**
EC	351.969	**< 0.001**	19.708	**< 0.001**	16.463	**< 0.001**
TN	66.917	**< 0.001**	12.811	**< 0.001**	6.841	**< 0.001**
TP	94.672	**< 0.001**	5.525	**0.021**	4.904	**0.003**
TOC	77.530	**< 0.001**	10.658	**0.002**	5.425	**0.002**
PC1	150.210	**0.001**	13.10	**< 0.001**	8.95	**< 0.001**
PC2	24.197	**< 0.001**	1.957	0.165	6.798	**< 0.001**
PC3	4.645	**0.004**	0.311	0.578	1.714	0.1689

Note: Asterisks values represent significant differences. S, soil substrate; M, microbial inoculants; Ew, earthworm. MWD, mean weight diameter; LAC, large aggregate content; EC, electrical conductivity; TN, total nitrogen; TP, total phosphorus; TOC, total organic carbon.

Table S4 Effects of soil substrates, microbial inoculants, and earthworms on soil physicochemical properties (Mean ± SD)

S	M	Ew	LAC (%)	MWD (mm)	EC (μS/cm)	TN (g/kg)	TP (g/kg)	TOC (%)
C	C	C	0.8186 ± 0.0426	1.1871 ± 0.1765	363.2 ± 55.3	1.118 ± 0.094	0.930 ± 0.022	1.574 ± 0.328
C	C	Ew	0.7567 ± 0.0764	0.9817 ± 0.2182	477.6 ± 99.8	1.241 ± 0.126	0.967 ± 0.025	1.414 ± 0.086
C	Bm	C	0.7370 ± 0.0505	1.0081 ± 0.1810	396.6 ± 47.1	1.520 ± 0.195	1.011 ± 0.018	1.714 ± 0.336
C	Bm	Ew	0.6998 ± 0.0623	0.8027 ± 0.1422	462.8 ± 90.2	1.216 ± 0.104	0.954 ± 0.049	1.422 ± 0.054
C	CB	C	0.7333 ± 0.0586	0.9049 ± 0.2359	401.3 ± 174.6	1.282 ± 0.103	0.941 ± 0.023	1.484 ± 0.164
C	CB	Ew	0.7447 ± 0.0741	0.9295 ± 0.2226	528.7 ± 78.5	1.370 ± 0.161	0.942 ± 0.056	1.487 ± 0.146
T1	C	C	0.8375 ± 0.0323	1.2988 ± 0.0624	309.6 ± 80.1	1.292 ± 0.177	0.974 ± 0.031	1.560 ± 0.324
T1	C	Ew	0.7647 ± 0.0255	1.1615 ± 0.2888	344.8 ± 63.6	1.240 ± 0.082	0.966 ± 0.037	1.378 ± 0.120
T1	Bm	C	0.8761 ± 0.0816	1.3417 ± 0.2042	256.1 ± 29.8	1.230 ± 0.147	0.977 ± 0.074	1.579 ± 0.247
T1	Bm	Ew	0.7993 ± 0.0546	1.1589 ± 0.1385	325.8 ± 42.1	1.128 ± 0.064	0.958 ± 0.024	1.352 ± 0.265
T1	CB	C	0.8177 ± 0.0085	1.1543 ± 0.1167	319.8 ± 20.7	1.442 ± 0.186	0.988 ± 0.049	1.675 ± 0.191
T1	CB	Ew	0.7728 ± 0.0514	1.0894 ± 0.1541	397.4 ± 24.9	1.175 ± 0.091	0.959 ± 0.033	1.271 ± 0.081
T2	C	C	0.8216 ± 0.0227	1.2906 ± 0.1303	2070.5 ± 160.6	5.084 ± 0.899	2.735 ± 0.189	6.216 ± 0.907
T2	C	Ew	0.7807 ± 0.0373	1.2315 ± 0.1919	1632.8 ± 370.4	2.611 ± 0.342	1.860 ± 0.287	3.672 ± 1.104
T2	Bm	C	0.7527 ± 0.0355	1.1950 ± 0.2006	1771.8 ± 259.9	4.490 ± 0.543	2.638 ± 0.256	6.372 ± 0.790
T2	Bm	Ew	0.7516 ± 0.0532	1.2539 ± 0.2164	1222.4 ± 166.0	3.198 ± 1.175	2.067 ± 0.336	4.367 ± 1.626
T2	CB	C	0.7483 ± 0.0430	1.2426 ± 0.1224	1916.8 ± 348.6	4.354 ± 0.570	2.245 ± 0.257	4.981 ± 0.803
T2	CB	Ew	0.7409 ± 0.0354	1.1423 ± 0.0829	1430.8 ± 96.7	2.894 ± 0.941	1.943 ± 0.428	3.563 ± 1.499
T3	C	C	0.6748 ± 0.0826	0.8113 ± 0.1665	1504.0 ± 117.4	3.972 ± 2.193	2.140 ± 0.662	3.998 ± 1.694
T3	C	Ew	0.7229 ± 0.0649	1.0151 ± 0.1649	1358.8 ± 232.6	3.297 ± 1.410	2.112 ± 1.024	4.092 ± 1.750
T3	Bm	C	0.6262 ± 0.0761	0.8406 ± 0.0966	1640.2 ± 180.0	2.822 ± 1.327	1.989 ± 0.737	3.863 ± 2.009
T3	Bm	Ew	0.7158 ± 0.0444	0.9500 ± 0.1506	1147.0 ± 113.3	2.502 ± 0.377	1.781 ± 0.375	3.155 ± 0.645
T3	CB	C	0.6391 ± 0.0175	0.7682 ± 0.0430	1467.5 ± 304.5	2.868 ± 0.954	1.919 ± 0.510	3.374 ± 1.479
T3	CB	Ew	0.7221 ± 0.0506	0.9772 ± 0.1004	1204.6 ± 125.0	3.025 ± 0.862	2.131 ± 0.465	3.621 ± 1.095

Note: Soil substrates(S): control(C), coal gangue-incorporated soil (T1), organic fertilizer-incorporated soil. (T2), and organic fertilizer surface-applied soil (T3). Microbial inoculants(M): control (C), *Bacillus megaterium* (Bm) and a compound *Bacillus* (CB). Earthworm (Ew): control(C), Ew. MWD, mean weight diameter; LAC, large aggregate content; EC, electrical conductivity; TN, total nitrogen; TP, total phosphorus; TOC, total organic carbon.

Table S5 Three-way ANOVA (partial η^2) results for the effects of soil substrates , microbial inoculants , and earthworms on soil structure and physicochemical properties.

Trait	partial η^2 (S)	partial η^2 (M)	partial η^2 (Ew)	partial η^2 (S*M)	partial η^2 (S*Ew)	partial η^2 (M*Ew)	partial η^2 (S*M *Ew)
MWD	0.47	0.06	0.01	0.05	0.14	0.01	0.04
LAC	0.47	0.08	0.01	0.12	0.23	0.03	0.01
EC	0.93	0.08	0.21	0.15	0.40	0.03	0.04
TN	0.70	0.01	0.13	0.07	0.19	0.01	0.04
TP	0.76	0.01	0.06	0.07	0.14	0.01	0.03
TOC	0.72	0.02	0.11	0.06	0.15	0.01	0.02

Note: Soil substrates(S). Microbial inoculants(M). Earthworm (Ew). MWD, mean weight diameter; LAC, large aggregate content; EC, electrical conductivity; TN, total nitrogen; TP, total phosphorus; TOC, total organic carbon.

Table S6 Principal Component Analysis of the Effects of Soil Substrates and Earthworm Addition on the Structure and Nutrient Properties of Soil

Trait	PC1	PC2	PC3
MWD	0.019	-0.724	0.162
LAC	0.187	-0.671	-0.098
EC	-0.465	-0.025	0.854
TN	-0.498	-0.091	-0.325
TP	-0.503	-0.052	-0.207
TOC	-0.497	-0.113	-0.293

Note: MWD, mean weight diameter; LAC, large aggregate content; EC, electrical conductivity; TN, total nitrogen; TP, total phosphorus; TOC, total organic carbon.
